# Supplementary material for: The interactome of intact mitochondria by cross-linking mass spectrometry provides evidence for coexisting respiratory supercomplexes
Source: Mol Cell Proteomics. 2017 Dec 8;17(2):216–32. doi: 10.1074/mcp.RA117.000470 (PMC5795388; doi:10.1074/mcp.RA117.000470)
Supplement: Supplemental Data [file supp_RA117.000470_133922_0_supp_23978_fzffwf.zip › spectra_annotation/mito_spectra_annotation/47-1-8-1-3-1.pdf]

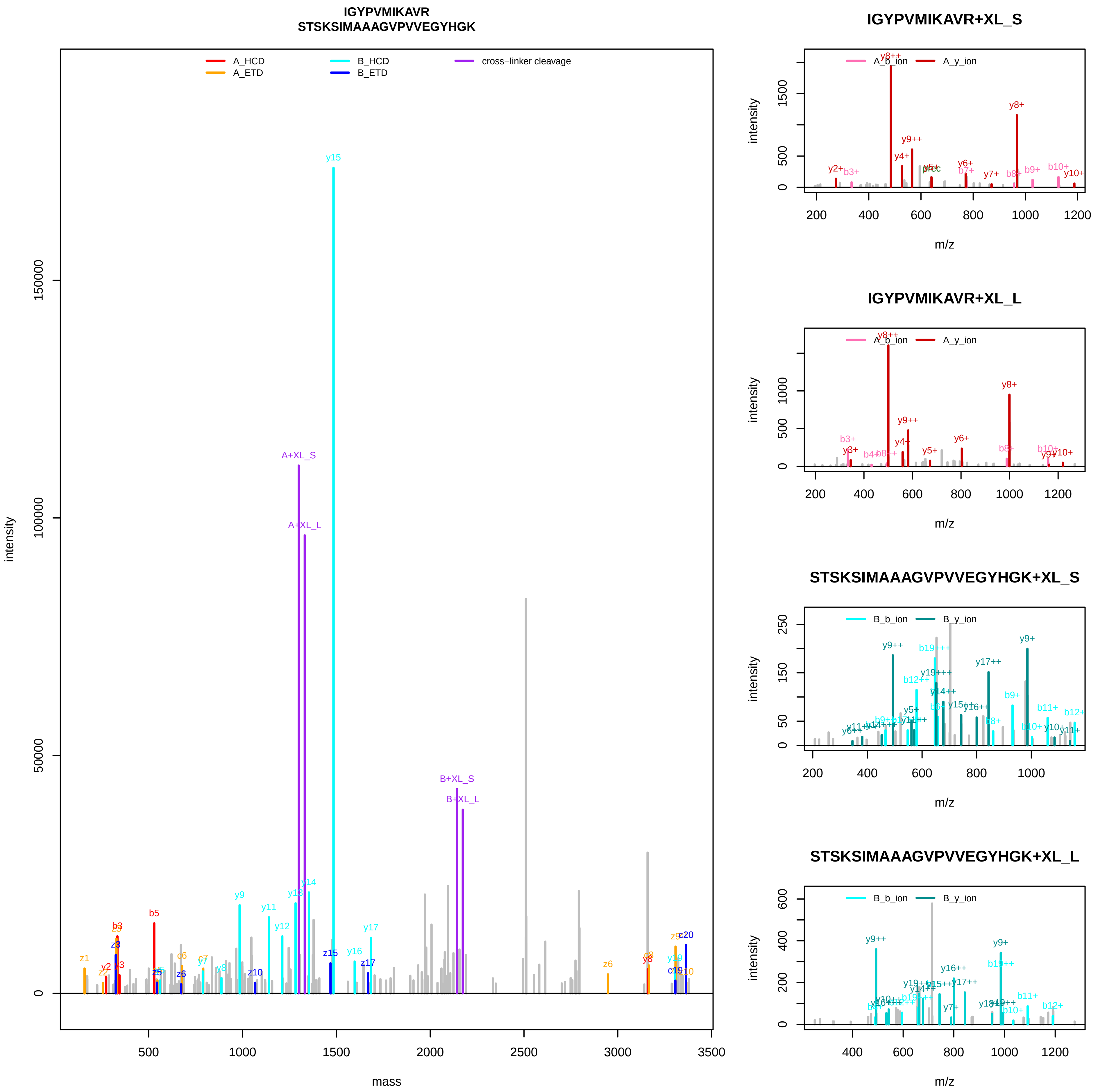
IGYPVMIKAVR
STSKSIMAAAGVPVVEGYHGK
A_HCD
A_ETD
B_HCD
B_ETD
cross−linker cleavage
A+XL_S
A+XL_L
B+XL_S
B+XL_L
y15
y14
y13
y12
y11
y9
y8
y7
y16
y17
y19
b5
b3
z1
z2
z3
z5
z6
z10
z15
z17
z9
c6
c7
c19
c20
intensity
mass
0
50000
100000
150000
500
1000
1500
2000
2500
3000
3500
IGYPVMIKAVR+XL_S
IGYPVMIKAVR+XL_L
STSKSIMAAAGVPVVEGYHGK+XL_S
STSKSIMAAAGVPVVEGYHGK+XL_L
A_b_ion
A_y_ion
B_b_ion
B_y_ion
m/z
y8++
y8+
y9++
y4+
y2+
b3+
y5+
y6+
b7+
y7+
b8+
b9+
b10+
y10+
y9+
y17++
y16++
y15++
y14++
b12++
b19++
y19+++
b11+
b12+
b10+
y11+
y7+
